# High prevalence of carbapenem resistance and clonal expansion of *bla*_*NDM*_ gene in *Klebsiella pneumoniae* isolates in an Iranian referral pediatric hospital

**DOI:** 10.1186/s13099-024-00611-1

**Published:** 2024-03-28

**Authors:** Babak Pourakbari, Setareh Mamishi, Shiva Poormohammadi, Reihaneh Hosseinpour Sadeghi, Shima Mahmoudi

**Affiliations:** 1 Pediatric Infectious Disease Research Center, Tehran University of Medical Sciences, Tehran, Iran; 2 Department of Infectious Diseases, Pediatrics Center of Excellence, Children’s Medical Center, Tehran University of Medical Sciences, Tehran, Iran; 3 Present Address: Biotechnology Centre, Silesian University of Technology, Gliwice, 44-100 Poland; 4 Tehran University of Medical Sciences, Tehran, Iran

**Keywords:** *Klebsiella pneumoniae*, Genotyping, Carbapenem resistance, *bla*_*NDM*_

## Abstract

**Background:**

The increasing global concern regarding antibiotic resistance necessitates in-depth studies to comprehend the phenotypic and genotypic characteristics of resistant bacterial strains. This study aimed to investigate the prevalence, antibiotic resistance profiles, and molecular characteristics of carbapenem-resistant *Klebsiella pneumoniae* (CRKP) isolates in an Iranian referral pediatrics hospital. Methods: In this study, we examined CRKP isolates collected from hospitalized pediatric patients across various wards. The isolates underwent antimicrobial susceptibility testing, the polymerase chain reaction (PCR) analysis for carbapenemase genes (*bla*_*NDM*_, *bla*_*VIM*_ and *bla*_*IMP*_), and genetic relatedness assessment using pulsed-field gel electrophoresis (PFGE).

**Results:**

Among 166 *K. pneumoniae* isolates, 54 (32.5%) exhibited resistance to carbapenems. Notably, all these resistant isolates were resistant to imipenem, with 35 (65%) displaying resistance to both imipenem and meropenem. Of the 54 CRKP isolates, 24 (44%) were metallo-β-lactamases (MBL)-producing. The prevalence of the *bla*_*NDM*_ gene among CKCP and MBL-producing isolates was 20% (*n* = 11) and 44% (*n* = 24), respectively. The *bla*_*VIM*_ and *bla*_*IMP*_ genes were not detected in any of the isolates. Twenty-six CRKP isolates (48%) were recovered from ICUs. PFGE analysis of CRKP isolates revealed 20 clusters, with cluster S being the most prevalent, comprising 24% of the total (*n* = 13).

**Conclusion:**

Our study reveals a concerning prevalence of carbapenem resistance in *K. pneumoniae* isolates. Specifically, the detection of the *bla*_*NDM*_ gene in 20% of CRKP isolates, with a significant proportion (82%) observed in isolated CRKP from the ICUs and emergency departments, underscores the potential clonal expansion of these resistant strains within these critical hospital wards.

## Background

Carbapenem-resistant *Klebsiella pneumoniae* (CRKP) poses a significant challenge in healthcare, presenting a substantial threat due to its resistance to essential antibiotics [1–4]. The increasing prevalence of CRKP is particularly noteworthy within healthcare settings, where infections caused by these strains are linked to limited treatment options and heightened patient morbidity and mortality. The implications extend beyond individual patient outcomes to encompass broader concerns related to infection control, antimicrobial stewardship, and public health [5].

The predominant mechanism of carbapenem resistance in carbapenem-resistant Enterobacteriaceae (CRE) involves the production of carbapenemase enzymes. Notable enzymes include those belonging to class A, such as *Klebsiella pneumoniae* carbapenemases (KPC), class B metallo-β-lactamases (MBL) like New Delhi MBL (NDM), verona integron-encoded MBL (VIM), and Imipenemase (IMP), and class D enzymes such as oxacillinases (OXA-48) [4]. In recent years in Iran, there has been an emergence of OXA-48 and NDM-1 producing *K. pneumoniae* strains associated with distinct clones, particularly ST11, ST893, and ST147 [6, 7]. The rising prevalence of MBL-producing gram-negative isolates, particularly strains of *K. pneumoniae* carrying the *bla*_*NDM*_ gene, in our hospitals is becoming a significant cause for concern [8]. This trend signifies a growing challenge in the management of bacterial infections, as MBLs are enzymes capable of hydrolyzing a broad range of beta-lactam antibiotics, including carbapenems. The spread of these resistant strains could potentially lead to limited treatment options, increased mortality rates, prolonged hospitalization, and higher healthcare costs. Addressing this concern requires a comprehensive approach, including enhanced surveillance, infection control measures, antibiotic stewardship, and continued research to understand the epidemiology and mechanisms of resistance. In response to this concern, we have decided to initiate a genotyping study employing Pulsed-Field Gel Electrophoresis (PFGE) specifically for CRKP isolates within our hospital. This initiative is crucial for enhancing our comprehension of the local dynamics of CRKP strains, enabling us to implement targeted measures for infection control and contribute to a more effective management strategy. The main objective of this study was to conduct genotyping on CRKP isolates collected over the course of one year using the PFGE method at Children’s Medical Center (CMC), Tehran, Iran.

## Method

### Study design

The study was conducted at the CMC, known as Iranian leading hospital for pediatric healthcare, education, and research between April 2021 and March 2022. With an extensive capacity of over 300 beds, CMC hosts more than 20 specialty and subspecialty wards. The Intensive Care Unit (ICU) facilities at CMC Hospital include the Pediatric Intensive Care Unit (PICU), the Neonatal Intensive Care Unit (NICU) for newborn infants, NICU OH for neonates following open heart and cardiac surgery, cardiac intensive care unit (CICU), and the infant ICU (IICU). The emergency ward, equipped with 40 beds, serves as a major referral center in the country, complemented by a 9-bed ICU.

This study was approved by the Ethics Committee of Tehran University of Medical Sciences, Tehran, Iran (IR.TUMS.CHMC.REC.1398.109).

### Bacterial identification and antibiotic susceptibility testing

Identification of *K. pneumoniae* was performed by using conventional biochemical methods. Preliminary identification of bacteria was done based on colony characteristics of grown isolates on media. Various biochemical tests were employed to identify *K*. *pneumoniae* isolates, including triple sugar iron agar, indole, motility, hydrogen sulfide production, citrate utilization, urease production, and lysine decarboxylase tests. All isolates were stored at -80 °C in tryptic soy broth (Merk, Germany) and 15% glycerol for further molecular studies.

The antimicrobial susceptibility of *K. pneumoniae* isolates was assessed using the Kirby-Bauer disk diffusion method on Mueller Hinton agar (Merck, Germany). Antimicrobial agents that were tested are as follows: ciprofloxacin (5 µg), cefepime (30 µg), cefotaxime (30 µg), amikacin (30 µg), gentamcin (10 µg), piperacillin/tazobactam (110/10 µg), colistin (50 µg), trimethoprim-sulfamethoxazole (1.25/23.75 µg), and nitrofurantoin (300 µg). The results were interpreted according to the guidelines recommended by the Clinical and Laboratory Standards Institute (CLSI) [9]. Minimum inhibitory concentrations (MICs) for meropenem and imipenem were determined using E-test, and the interpretations were based on breakpoints provided by CLSI [9]. The antibiotics were obtained from MAST, UK. *E. coli* ATCC 25,922 and *Pseudomonas aeruginosa* ATCC 27,853 were used as quality control strains. CRKP clinical isolates were recognized by resistant to at least one of the carbapenem (imipenem, meropenem) based on CLSI guidelines.

We used the double disk synergy test (DDST) for the phenotypic identification of MBL-producing isolates [8].

### Polymerase chain reaction (PCR) assays

The DNA of all CRKP strains was extracted using the DNA extraction kit (GeNet Bio Company, Daejeon, Korea) according to the manufacturer’s instruction. Following extraction, PCR analysis was conducted targeting the *bla*_*NDM*_, *bla*_*VIM*_ and *bla*_*IMP*_ genes using specific primers as previously described [8].

### PFGE

Genetic relationships of the CRKP isolates were assessed using PFGE. The genomic DNA was embedded in 1% agarose blocks and then subjected to enzymatic digestion with 50 U Fast XbaI restriction enzyme for 30 min. Subsequently, the DNA fragments were separated using the CHEF-DR II system (Bio-Rad Laboratories), employing 1% agarose (SeaKem Gold®) gel at a voltage of 6 V/cm. The electrophoresis parameters included an initial pulse duration of 2.2 s, a final pulse duration of 63.8 s, and a running temperature of 14 °C for a total duration of 22 h. The standard strain *Salmonella enterica* serotype Braenderup H9812 was employed as a reference [10].

The electrophoretic patterns obtained were analyzed through visual inspection to detect the position and number of bands. The DNA pattern was analyzed using Gelcompar II version 6.5 software (Applied Maths, Belgium). A similarity coefficient was determined using Dice coefficients, followed by cluster analysis using the unweighted pair group method with arithmetic averages (UPGMA). Isolates displaying a similarity cut-off of ≥ 80% in their banding patterns were classified as belonging to the same clonal lineage.

### Statistical analysis

Statistical analyses were carried out using IBM SPSS Statistics (version 23.0, IBM Corp. Armonk, NY, USA). Descriptive statistics such as mean, percentage and frequency were used to indicate categorical data. The variables underwent evaluation through the chi-square test. In accordance with this study, statistical significance was defined as a p-value of ≤ 0.05.

## Result

A total of 166 isolates of *K. pneumoniae* were collected from patients, with 54 (32.5%) identified as CRKP. Among the 54 CRKP isolates, 65% (*n* = 35) were obtained from male. The median age of the patients was 2.6 months (interquartile range: 1 month- 4.2 years). The mean duration of hospital stay was 4.4 ± 3.5 days.

The samples yielding the highest number of isolates were urine, with twenty cases (37%), followed by blood, which produced fifteen isolates (28%). Next in frequency were isolates from bronchoalveolar lavage (BAL), accounting for five cases (9%), and wounds, contributing three cases (6%). Eye discharge yielded two isolates (4%), while throat, cerebrospinal fluid (CSF), sputum, and dialysis fluid, each resulted in one isolate (2%). An additional five isolates (10%) were obtained from other sources. The PICU had the highest number of CRKP isolates at 19% (*n* = 10), followed by emergency with 17% (*n* = 9) and NICU with 13% (*n* = 7). Surgery 1 and NICU OH comprised 9% (*n* = 5) and 7% (*n* = 4) of the isolates, respectively, while nephrology, neonatal, and IICU each contributed 6% (*n* = 3). CICU, cardiopulmonary, gastroenterology, and urology each accounted for 4% (*n* = 2), while surgery 2 and neurology had the lowest representation at 2% (*n* = 1) each.

In this study, 32.5% of *K. pneumoniae* isolates demonstrated carbapenem resistance. The antibiotic resistance pattern observed in the isolates of CKCP was as follows: imipenem (54 out of 54, 100%), cefotaxime (49 out of 54, 91%), cefepime (45 out of 54, 83%), piperacillin-tazobactam (43 out of 54, 80%), nitrofurantoin (14 out of 18, 74%), amikacin (39 out of 54, 72%), trimethoprim-sulfamethoxazole (35 out of 54, 65%), meropenem (35 out of 54, 65%), ciprofloxacin (11 out of 19, 58%), gentamicin (29 out of 54, 54%), and colistin (2 out of 15, 13%). DDST results showed that 24 CKCP isolates (44%) were MBL-producing ones. The prevalence of the *bla*_*NDM*_ gene among the CKCP and MBL-producing isolates was 20% (*n* = 11) and 44% (*n* = 24), respectively. The *bla*_*VIM*_ and *bla*_*IMP*_ genes were not detected in any of the isolates.

**Fig. 1:**
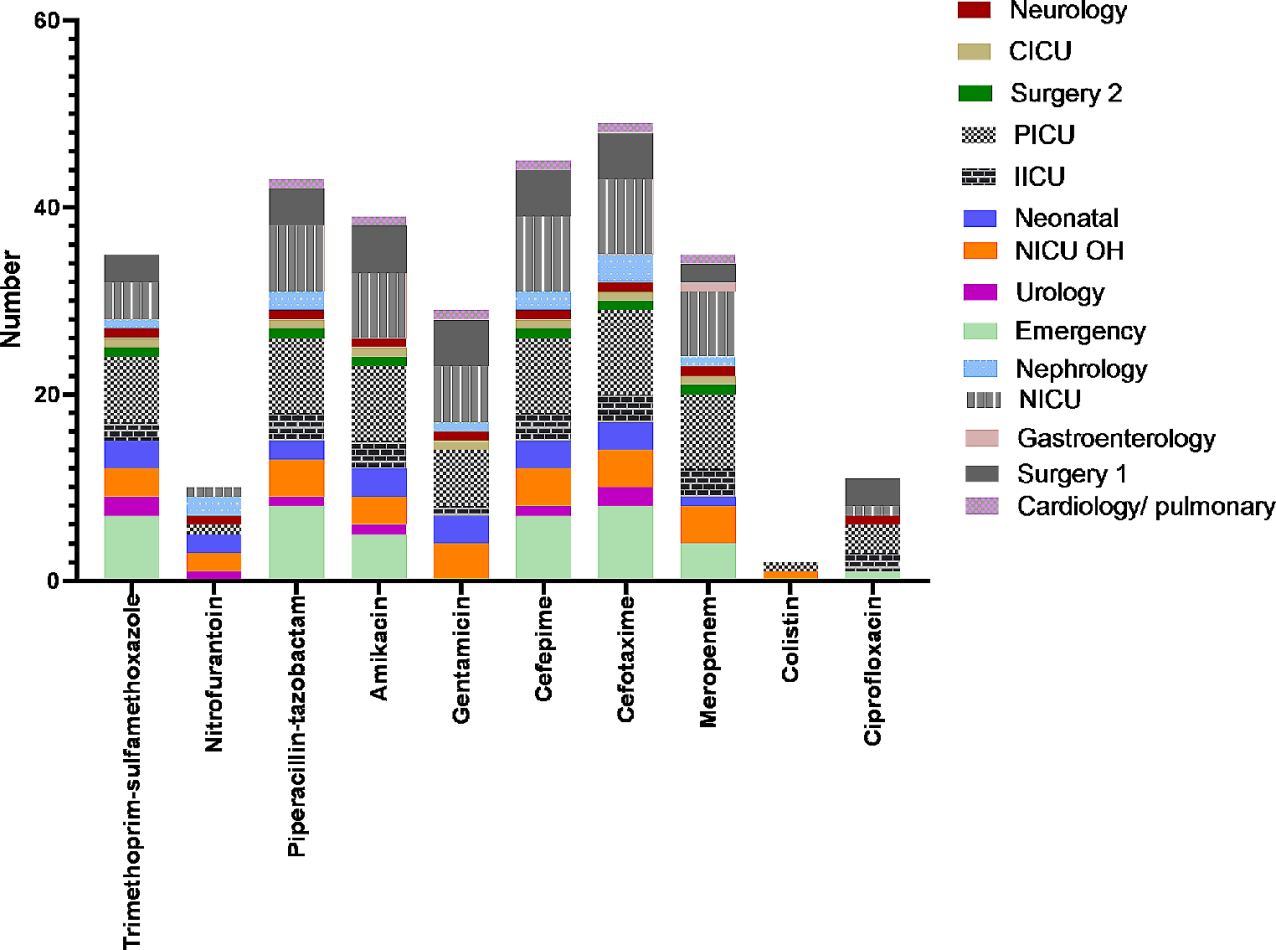
Antimicrobial resistance patterns of the CRKP isolates obtained from patients across various hospital wards

Figure 1 illustrates the antimicrobial resistance of CKCP isolates across various wards. In the PICU and NICU, antibiotic resistance rates were notably high.

**Fig. 2:**
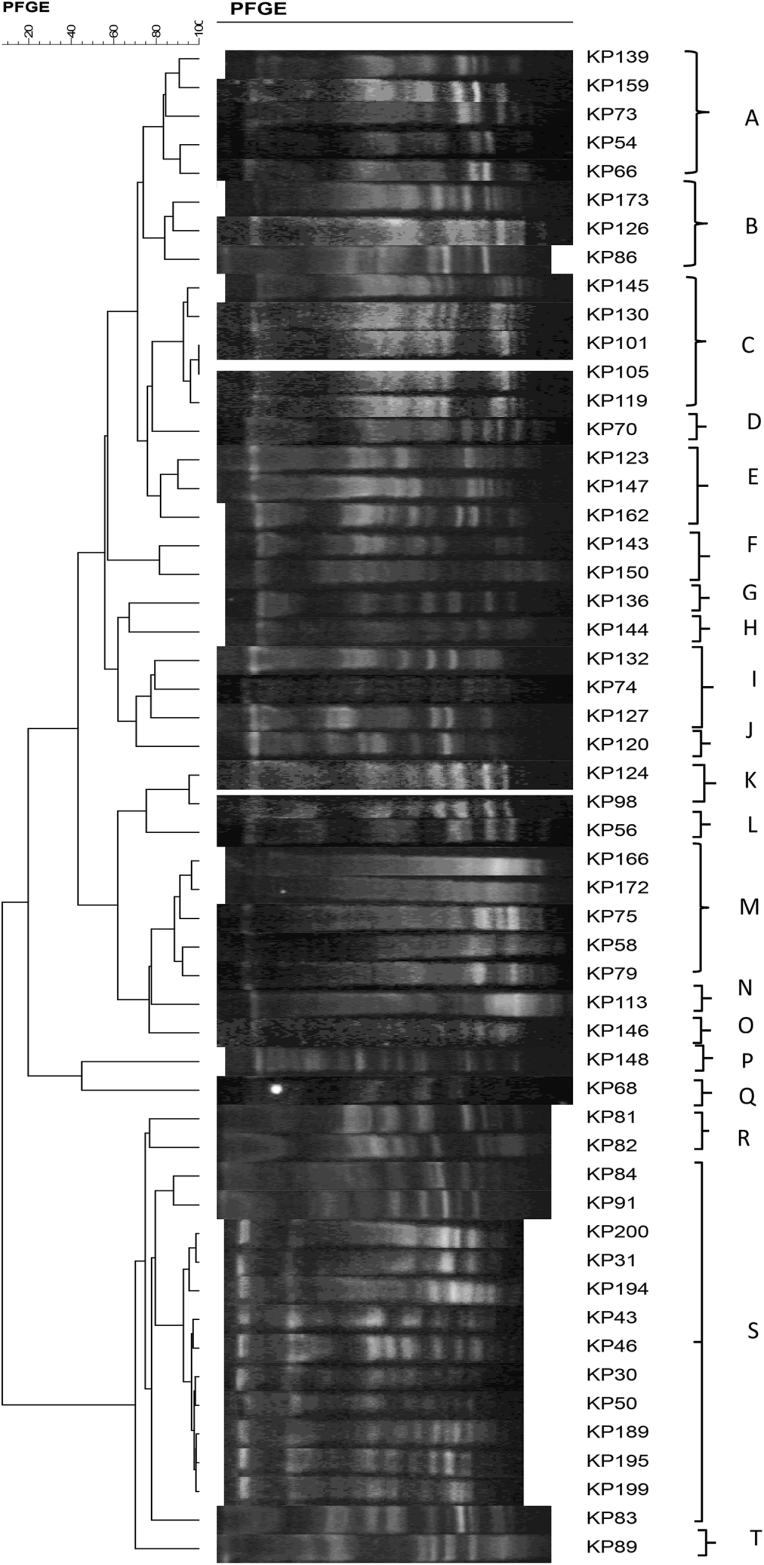
Dendrogram of PFGE typing of 53 CRKP isolates, cluster classification based on ≥ 80% similarity genotypes

PFGE analysis of the CRKP isolates identified 20 clusters, with cluster S being the most prevalent, comprising 24% of the total (*n* = 13) (Fig. 2). Following, clusters A, C, and N each accounted for 9% of the isolates, with five samples in each cluster. Cluster B and J represented 6% of the isolates, with three samples each. The distribution of different clusters across various hospital wards illustrates varied patterns of occurrence (Table 1). Cluster A was predominantly found in the NICU, representing 60% of occurrences. Cluster B was primarily observed in the PICU, constituting 67% of occurrences. Cluster C was distributed across multiple wards, with 40% of occurrences in PICU and minor presence in IICU, NICU OH, and emergency wards. Cluster D was solely identified in the neonatal ward. Cluster E exhibited an equal distribution across urology, cardiopulmonary, and surgery 1 wards. Cluster J and cluster K were solely observed in the emergency and PICU, respectively. Cluster L was solely present in NICU. Cluster M was distributed across nephrology, surgery 1, emergency, and NICU wards. Cluster S was predominantly found in the emergency ward (23%) and also observed in the CICU and PICU (each 15%). It appears in other wards as well, including urology, surgery 2, nephrology, and cardiopulmonary wards.

**Table 1:** The distribution of CRKP isolates in CMC hospital

Cluster	Number of genotypes	Ward (n, %)	Sample type (n, %)	*bla*_*NDM*_ (n, %)
A	5	NICU (3, 60)	Urine (3, 60)	1 (20)
		Neonatal (1, 20)	Eye discharge (1, 20)	
		NICU OH (1, 20)	Blood (1, 20)	
B	3	PICU (2, 67)	Blood (1, 33)	1 (33)
		IICU (1, 33)	BAL (2, 67)	
C	5	PICU (2, 40)	Blood (2, 40)	4 (80)
		IICU (1, 20)	Urine (2, 40)	
		NICU OH (1, 20)	Dialysis fluid (1, 20)	
		Emergency (1, 20)		
D	1	Neonatal (1, 100)	Urine (1, 100)	0
E	3	Urology (1, 33)	Urine (1, 33)	1 (33)
		Cardiopulmonary (1, 33)	Sputum (1, 33)	
		Surgery 1 (1, 33)	Blood (1, 33)	
F	2	Neurology (1, 50)	Urine (1, 50)	0
		Surgery 1 (1, 50)	BAL (1, 50)	
G	1	PICU (1, 100)	Blood (1, 100)	0
H	1	NICU OH (1, 100)	Other (1, 100)	0
I	3	NICU OH (1, 33)	Blood (2, 67)	0
		NICU (1, 33)	Other (1, 33)	
		Gastroenterology (1, 33)		
J	1	Emergency (1, 100)	Blood (1, 100)	0
K	2	PICU (2, 100)	Blood (1, 50)	2 (100)
			Wounds (1, 50)	
L	1	NICU (1, 100)	Urine (1, 100)	0
M	5	Nephrology (1, 20)	Urine (2, 40)	0
		Surgery 1 (2, 40)	BAL (1, 20)	
		Emergency (1, 20)	Eye discharge (1, 20)	
		NICU (1, 20)	CSF (1, 20)	
N	1	Gastroenterology (1, 100)	Other (1, 100)	0
O	1	Surgery 1 (1, 100)	Wounds (1, 100)	1 (100)
P	1	PICU (1, 100)	Other (1, 100)	0
Q	1	Emergency (1, 100)	Urine (1, 100)	0
R	2	NICU (1, 50)	Wounds (1, 50)	0
		Nephrology (1, 50)	Urine (1, 50)	
S	13	CICU (2, 15)	Urine (7, 54)	0
		Emergency (3, 23)	Throat (1, 8)	
		Ourology (1, 8)	Blood (4, 30)	
		Surgery 2 (1, 8)	Other (1, 8)	
		Nephrology (1, 8)		
		Cardiopulmonary (1, 8)		
		PICU (2, 15)		
		IICU (1, 8)		
		Neonatal (1, 8)		
T	1	Emergency (1, 100)	Blood (1, 100)	0
Non-typable	1	Emergency (1, 100)	BAL (1, 100)	1 (100)

**Fig. 3:**
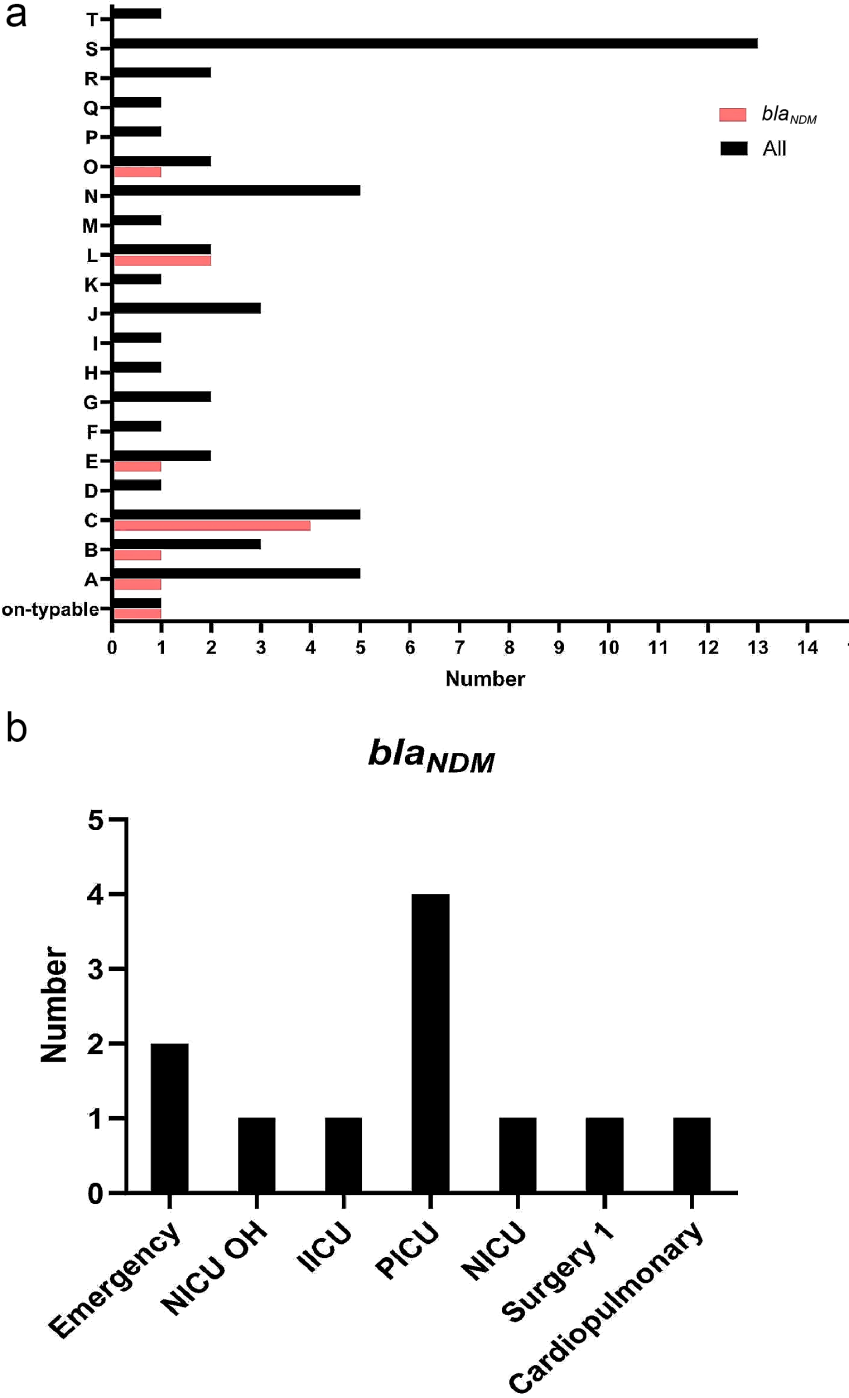
The distribution of CRKP isolates harboring the *bla*_*NDM*_ gene; (**a**) by cluster, (**b**) by wards

Figure 3a illustrates the distribution of *bla*_*NDM*_ -positive CRKP isolates. Cluster C exhibited the highest occurrence, with four samples identified as *bla*_*NDM*_ -positive CRKP isolates. Clusters L accounted for two *bla*_*NDM*_ -positive CRKP isolates. Additionally, Clusters A, B, E, and O each contributed one *bla*_*NDM*_-positive CRKP isolate. Furthermore, one *bla*_*NDM*_ -positive CRKP isolate was classified as non-typable.

The majority of *bla*_*NDM*_-positive CRKP isolates were observed in the PICU, constituting 36% of isolates. Additionally, the emergency ward accounted for 18% of occurrences, while NICU OH, IICU, NICU, Surgery 1, and cardiopulmonary wards each had 9% representation of *bla*_*NDM*_-positive CRKP isolates (Fig. 3b).

## Discussion

The emergence of CRKP presents a significant challenge to worldwide public health, posing a substantial obstacle to effective clinical infection management [4, 11–13]. This study sought to explore the drug resistance profile and epidemiological features of CRKP at the CMC, with the overarching goal of implementing effective measures to curb the spread of CRKP infections among pediatric patients. In this study, 32.5% of *K. pneumoniae* isolates demonstrated carbapenem resistance. According to the latest meta-analysis, the global prevalence of CRKP in patients with *K. pneumoniae* infections was 28.69% (95% CI: 26.53–30.86%) [14]. In a previous study conducted at CMC between January 2018 and April 2020, *K. pneumoniae* isolates showed resistance rates of 38% to imipenem and 20% to meropenem.

In our study, the prevalence of MBL-producing *K. pneumoniae* isolates was found to be 44%, aligning with previous findings [8]. Among CKCP and MBL-producing isolates, the prevalence of the *bla*_*NDM*_ gene was 20% and 83%, respectively. Notably, the prevalence of the *bla*_*NDM*_ gene surpassed previous reports, wherein only 42% of MBL-producing isolates tested positive for *bla*_*NDM*_ [8].

The high prevalence of CRKP complicates the management of nosocomial infections, underscoring the urgency for a reassessment of current infection control protocols and antibiotic stewardship strategies. The predominant risk factors commonly associated with CRE infection include the utilization of indwelling medical devices, previous exposure to antibiotics, and admission to the ICUs [4]. The substantial number of CRKP isolates, particularly isolated from urine (37%) and blood (28%), suggests a potential association with the utilization of indwelling medical devices. Urinary catheters and central venous catheters, frequently utilized in healthcare settings to address diverse medical needs and ensure critical patient care, establish an optimal environment for bacterial colonization. Moreover, prior antibiotic exposure is identified as another key risk factor driving the emergence and spread of CRE infections [15]. All patients included in our study had a history of previous hospitalization and prior antibiotic use. Enhanced infection control measures, including stringent adherence to hand hygiene protocols, judicious use of indwelling medical devices, and implementation of antimicrobial stewardship programs to optimize antibiotic use, are essential for controlling the spread of CRE.

Twenty-six CRKP isolates (48%) were obtained from ICUs, aligning with findings from other studies that have consistently reported the highest incidence of CRKP infections among patients admitted to ICU wards [16, 17]. According to the latest meta-analysis, the prevalence of hospital-acquired CRKP infections is notably high in ICUs, with a pooled estimate of 62.31% [14]. ICUs, characterized by high patient acuity, prolonged hospital stays, and frequent invasive procedures, provide a conducive environment for the transmission of healthcare-associated pathogens like CRE [18]. Furthermore, the compromised immune status of ICU patients, coupled with the frequent use of broad-spectrum antibiotics and invasive medical interventions, further heightens their susceptibility to CRE infections.

All CRKP isolates in our study were multidrug-resistant and showed high resistance to piperacillin-tazobactam and cephalosporins. The prevalence of resistance to colistin, and ciprofloxacin was low, which may be because of limited use of these antibiotics in children. This suggests that ciprofloxacin or colistin, in conjunction with other antibiotics such as meropenem and imipenem, may be useful for the treatment of complex CRKP infections.

Multiple outbreaks of CRKP infections carrying *bla*_*NDM*_ genes have been reported in Iran [7, 19–22]. Research conducted in Turkey, Egypt, and Greece, examining the PFGE patterns of clinical CRKP isolates, has provided significant evidence supporting the transmission and prevalence of resistance within hospital wards, particularly in ICU settings [23–25]. In the investigation conducted by Tao et al. in China, the PFGE analysis of the 86 CRKP strains demonstrated their segregation into 15 separate clusters, primarily found within neonatal medicine, neonatal ward and ICU [26]. In the study conducted by Celikbilek et al. in Turkey, PFGE analysis revealed that over half of the CRKP isolates were grouped into clusters. However, cross-transmission was not limited to a specific wards or time period [25].

The current study also demonstrated that cross-transmission was not restricted to a single ward. For instance, despite the 13 CRKP isolates being clustered within the largest PFGE type, they were identified across various wards, including the CICU, emergency, urology, surgery, nephrology, cardiopulmonary, PICU, IICU, and neonatal department. Four CRKP isolates observed in cluster C harbored the *bla*_*NDM*_ gene and were isolated from different ICU wards and emergency department. A significant association was observed between cluster C and the presence of the *bla*_*NDM*_ gene. The lack of adequate infection control measures in our hospital raise concerns regarding the potential spread of CRKP through patient transfers, particularly from the emergency department to other wards. The detection of the *bla*_*NDM*_ gene in other clusters implies the dissemination of this resistance gene across different wards within the hospital, thereby indicating the possibility of transmission to all wards. This clonal expansion has the potential to be transmitted to patients via personnel, equipment, or contaminated surfaces [25]. Therefore, it is highly recommended to enforce rigorous infection control protocols, encompassing effective isolation measures, thorough cleaning and disinfection protocols, and strict compliance with hand hygiene practices, to control the dissemination of CRKP within the hospital setting. Effective communication and collaboration among healthcare teams are vital to ensure that patients with CRKP colonization or infection are appropriately managed during transfers, thus minimizing the risk of further dissemination [5, 27].

While the *bla*_*NDM*_ gene is a significant contributor to carbapenem resistance, there are other mechanisms, such as the production of carbapenemases like KPC and OXA enzymes, as well as non-enzymatic mechanisms involving alterations in porins and efflux pumps, that can also confer resistance to carbapenems. Understanding the full spectrum of resistance mechanisms is essential for developing comprehensive strategies to combat carbapenem-resistant infections effectively.

The study faced a limitation in its capacity to differentiate between colonization and infection with CRKP. An in-depth analysis of the definition of nosocomial infections could have provided a stronger foundation for the study and improved the interpretation of its findings. **Conclusion**.

In conclusion, our study revealed a concerning prevalence of carbapenem resistance among *K. pneumoniae* isolates. Specifically, the detection of the *bla*_*NDM*_ gene in 20% of CRKP isolates, with a significant proportion (82%) observed in isolated CRKP from the ICUs and emergency departments, underscores the potential clonal expansion of these resistant strains within these critical hospital wards.

## Data Availability

The data that support the findings of this study are available from the corresponding author upon reasonable request.

## Abbreviations

CRKP: Carbapenem-resistant *Klebsiella pneumoniae*
CRE: Carbapenem-resistant Enterobacteriaceae
KPC: *Klebsiella pneumoniae* carbapenemases
MBL: Metallo-β-lactamases
NDM: New Delhi MBL
VIM: Verona integron-encoded MBL
IMP: Imipenemase
OXA: Oxacillinases
PFGE: Pulsed-field gel electrophoresis
CRKP: Carbapenem-resistant *Klebsiella pneumoniae*
ICU: Intensive care unit
PICU: Pediatric intensive care unit
NICU: Neonatal intensive care unit
NICU OH: Neonatal intensive care unit following open heart and cardiac surgery
IICU: Infant ICU
CICU: Cardiac intensive care unit
CMC: Children’s Medical Center
CLSI: Clinical and Laboratory Standards Institute
UPGMA: Unweighted pair group method with arithmetic averages
MICs: Minimum inhibitory concentrations
DDST: Double disk synergy test
BAL: Bronchoalveolar lavage
CSF: Cerebrospinal fluid
